# Short term treatment with a cocktail of rapamycin, acarbose and phenylbutyrate delays aging phenotypes in mice

**DOI:** 10.1038/s41598-022-11229-1

**Published:** 2022-05-04

**Authors:** Zhou Jiang, Juan Wang, Denise Imai, Tim Snider, Jenna Klug, Ruby Mangalindan, John Morton, Lida Zhu, Adam B. Salmon, Jackson Wezeman, Jiayi Hu, Vinal Menon, Nicholas Marka, Laura Neidernhofer, Warren Ladiges

**Affiliations:** 1 Department of Comparative Medicine, School of Medicine, University of Washington, Seattle, WA USA; 2 Biological Sciences Division, Pacific Northwest National Laboratory, Richland, WA USA; 3 Department of Pathology, Microbiology and Immunology, School of Veterinary Medicine, University of California, Davis, CA USA; 4 Department of Veterinary Pathobiology, College of Veterinary Medicine, Oklahoma State University, Stillwater, OK USA; 5 In Vivo Pharmacology, HD Bioscience Co., Ltd, Shanghai, China; 6 Department of Molecular Medicine, San Antonio Sam and Ann Barshop Institute for Longevity and Aging Studies, South Texas Veterans Health Care System, Geriatric Research Education and Clinical Center, The University of Texas Health Science Center at San Antonio, San Antonio, TX USA; 7 Department of Biochemistry, Molecular Biology, and Biophysics, Institute on the Biology of Aging and Metabolism, University of Minnesota, Saint Paul, MN USA; 8 Clinical and Translational Sciences Institute, Biostatistical Design and Analysis Center, University of Minnesota, Minneapolis, MN USA

**Keywords:** Ageing, Senescence, Experimental models of disease, Translational research, Diseases

## Abstract

Pharmaceutical intervention of aging requires targeting multiple pathways, thus there is rationale to test combinations of drugs targeting different but overlapping processes. In order to determine if combining drugs shown to extend lifespan and healthy aging in mice would have greater impact than any individual drug, a cocktail diet containing 14 ppm rapamycin, 1000 ppm acarbose, and 1000 ppm phenylbutyrate was fed to 20-month-old C57BL/6 and HET3 4-way cross mice of both sexes for three months. Mice treated with the cocktail showed a sex and strain-dependent phenotype consistent with healthy aging including decreased body fat, improved cognition, increased strength and endurance, and decreased age-related pathology compared to mice treated with individual drugs or control. The severity of age-related lesions in heart, lungs, liver, and kidney was consistently decreased in mice treated with the cocktail compared to mice treated with individual drugs or control, suggesting an interactive advantage of the three drugs. This study shows that a combination of three drugs, each previously shown to enhance lifespan and health span in mice, is able to delay aging phenotypes in middle-aged mice more effectively than any individual drug in the cocktail over a 3-month treatment period.

## Introduction

Aging is a complex process generally associated with multiple pathways^1,2^. Some of these pathways have been targeted by individual drugs resulting in increased lifespan or enhanced healthy aging in mice^3^. If multiple pathways of aging are involved in the aging process and the development of age-related diseases, then targeting more than one pathway should have additional benefits. One approach to accomplish this would be to develop combinations or cocktails of drugs that individually have been shown to attenuate the dysfunction and pathology seen with increasing age^4^. We selected a drug cocktail of rapamycin (Rap), acarbose (Acb), and phenylbutyrate (Pba) to test this concept in aging mice. The rationale for the drug cocktail is based on validated anti-aging effects of the individual drugs each targeting different but overlapping pathways of aging.

Rap is an antibiotic clinically approved for treating organ transplant patients^5,6^ and neoplastic conditions. It is orally bioavailable and readily crosses the blood brain barrier. Rap inhibits mTOR signaling which plays a significant role in integrating signals from growth factors and nutrients to control protein synthesis. The effect of down regulating mTOR on aging was confirmed by the Intervention Testing Program (funded by National Institute on Aging) showing that rapamycin extended lifespan in mice^7^. It has also been shown to delay age-associated heart disease and other pathologies in mice^8–10^. Transient Rap treatment has been shown to increase lifespan and healthspan in middle age mice^11^. The second drug in the cocktail, Acb, is a popular type 2 diabetes medication used for glucoregulatory control^12^. Considering that one of the hallmarks of caloric restriction (CR) is improved glucose homeostasis and insulin sensitivity^13^, the similarities between CR and Acb treatment underscore the potential metabolic responses that are present in conditions of improved aging. Like CR, Acb reduces body weight and body fat, improves glucose dysregulation associated with aging, and increases mouse lifespan^14^. The third drug in the cocktail, Pba, is a derivative of the short chain fatty acid butyrate, which occurs naturally in the gut. It is orally bioavailable and can be detected in the blood stream within 15 min of oral administration, and organs within an hour. We have shown that Pba can enhance physical and cognitive performance with increasing age in mice^15^.

Lifespan extension, which involves starting at a young age through end of life, has been a gold standard in testing anti-aging effects of drugs in preclinical animal models. While this approach provides valuable information, it has less translational value compared to studies starting at midlife for short periods of time with age-specific time points. These types of short-term studies have been reported in the mouse, a popular preclinical model for aging research. Middle-aged C57BL/6 (B6) mice are available from an NIH supported aging rodent colony for grantees funded by NIH so are a ready source of mice to use in short term drug intervention studies. The strain is well characterized in age-related phenotypes using physiological performance tests and geropathological assessment. Since testing a three-drug combination is a novel approach, a second strain would help validate drug effects on aging parameters seen in B6 mice. The HET3 4-way cross mouse strain is used exclusively by the Intervention Testing Program to test a large number of drugs, including the three drugs in our cocktail, in lifespan studies. Therefore, it would be a useful strain to help validate the effects of the cocktail on aging parameters in B6 mice.

In addition to using physiological performance tests as endpoints for aging parameters, we used an anatomic pathology-based assessment system designated as geropathology^10^. The geropathology system allows an in depth look at age-related pathological phenotypes in a quantitative-like manner. A grading platform has been established for mice, which generates lesion scores for individual organs thereby identifying which organs are more or less responsive to short-term drug treatment^16^. The system also identifies drug-responsive age-related lesions thus providing additional evidence of the impact of drug treatment on aging. A good example is inflammation, which is readily recognized and easily graded using routine stains of thin tissue sections on glass slides. Inflammation is considered one of the pathways of aging^1^ so would be of interest for further investigation in a drug study for the types of genes involved using transcriptomic and molecular applications.

We posited that the drug cocktail would be successful in delaying aging phenotypes because it would target not just inflammation, but more aging processes than any individual drug. For example, Rap targets autophagy and vascular deficits by downregulating mTOR signaling^17^. Acb indirectly targets insulin signaling, mitochondrial dysfunction and oxidative stress by blocking breakdown of complex carbohydrates in the small intestine so that less glucose is available for systemic absorption^18^. Pba targets dysfunctional proteostasis through the endoplasmic reticulum stress response and targets epigenetic function by inhibiting histone deacetylation resulting in upregulation of genes including anti-inflammatory genes^19,20^. We report here that the drug cocktail delays aging phenotypes in B6 and HET3 mice with several strain and sex dependent differences.

## Methods 

### Animals and drug treatment

C57BL/6 (B6) and HET3 4-way cross (HET3) mice of both sexes were used for this study. B6 mice were received from the NIA Aged Rodent Colony managed by Charles River, Inc., an approved specific pathogen free (SPF) vendor. HET3 mice were provided by the Nathan Shock Center at the University of Texas Health Science Center at San Antonio, TX, quarantined for four weeks and approved for specific pathogen free status before being housed in the general SPF mouse facilities. SPF status assures mice are free of common mouse pathogens by viral and bacterial tests done by an accredited laboratory animal diagnostic lab (IDEXX Bioanalytics). The SPF status of SPF rooms is monitored under the guidance of the Rodent Health Monitoring Program (RHMP) in the Department of Comparative Medicine at the University of Washington. The RHMP surveils and maintains SPF rodent housing integrity by routine monitoring, generally on a quarterly basis, and the quarantine and testing of incoming rodents from nonapproved vendors. Monitoring methods consist of collection of dust samples from exhaust plenums of individual ventilated cage (IVC) racks, and direct sampling from sentinel animals as needed. PCR analysis is done to detect infectious agents not acceptable in UW rodent SPF housing in concert with similar standards at Charles River and the NIA aging rodent colony. The following infectious agents are not allowed in mouse SPF rooms: mouse coronavirus, mouse parvovirus, minute virus of mice, reovirus‐3, pneumonia virus of mice, murine rotavirus, Theiler’s murine encephalitis virus, lymphocytic choriomeningitis virus, ectromelia, Sendai virus, Mycoplasma pulmonis, pinworms, fur mites, Helicobacter spp. and murine norovirus are excluded from selected rooms.

All mice were group housed (up to five per cage) in the new underground UW Health Sciences Complex 40,000 sq ft two-story Animal Research and Care Facility (ARCF) with structurally independent mechanical and HVAC systems allowing strict access and maintenance of specific pathogen free housing conditions. Animal housing rooms have a 14-h light/10-h dark cycle, average room temperature of 72 °F, humidity range of 30–70%, and ventilation of 10 to 15 fresh air exchanges per hour, all of which are checked monthly for proper function. Automatic watering provides reverse osmosis water with monthly flushing of water lines. Caging consisted of individual ventilated cages (IVC) in a ventilated rack system (NexGen Mouse 500, Allentown) of stainless steel and a combination of high-quality plastic easily sanitized and autoclavable, with quiet, energy efficient airflow motors, and a plastic runner system from cage to rack that allows health checks and other observations without fully removing the cage from the rack. Environmental enrichment consisted of shreddable paper products, for example nestlets, and polycarbonate tubes and huts on the cage floor for activity and escape venues. Bedding was autoclaved hardwood shavings, replaced biweekly.

Drugs were delivered orally in the chow (Labdiet 5LG6, see supplement for ingredients), which was prepared and irradiated by TestDiet, Inc (a division of Purina Mills, Richmond, IN) according to the same diet formula and procedures used by the Intervention Testing Program^15,21^. Five different medicated diets were prepared plus a non-medicated control diet. Doses for Rap, Acb and Pba were those that enhanced health span and lifespan as reported for mice^7,15,21^. Microencapsulated Rap was obtained from Southwest Research Institute (San Antonio, TX) and mixed in the chow at a concentration of 14 ppm. Acb was obtained from Spectrum Chemical Mfg Corp., Gardena, CA and mixed in the chow at a concentration of 1000 ppm. Pba was obtained from Triple Crown America, Inc, Perkasie, PA under the commercial name of sodium triButyrate, and mixed in the chow at a concentration of 1000 ppm. Control cohorts received LabDiet 5LG6 without any added medication (control chow). Before arriving at the UW, NIA mice had been on the NIH31 rodent chow (see supplement for ingredients), a diet similar to LabDiet 5LG6. HET3 mice had previously been on non-medicated LabDiet 5LG6 chow the same as the control chow and the same as used by the ITP Program. After arrival at the UW, all mice were acclimated to the same LabDiet 5LG6 control chow at least two weeks before randomly assigned to different drug treatment groups. Since the rodent diet used by the ITP Program was the same base diet with the same drug formulations used in our project, we had no concerns about changes in bioavailability of any of the drugs. In addition, we included HET3 mice, a strain used exclusively by the ITP Program, to help validate effects seen in C57BL/6 mice, a strain not used by the ITP Program.

Mice were 20 months of age at the start of the study, which continued for three months, when the study ended at 23 months of age. Each cohort had 28 mice, 14 females and 14 males. Females were tested first in staggered groups followed by males in staggered groups. The cohorts for B6 mice of each sex were designated as Control, Full-dose Cocktail, Half-dose Cocktail, Rapamycin, Acarbose, and Phenylbutyrate. The cohorts for HET3 mice of each sex were designated as Control and Full-dose Cocktail. All experiments using animals were approved by the University of Washington Institutional Animal Care and Use Committee. All procedures using animals, including euthanasia, were carried out in accordance with relevant guidelines and regulations and are reported in accordance with ARRIVE guidelines.

Mice were weighed weekly. Body fat mass and lean muscle mass were recorded monthly using a calibrated quantitative magnetic resonance imaging system (EchoMRI). Food consumption was monitored once a month over a three-day period wherein food weight difference was compared between 0 and 72 h. The amount of food consumed by each mouse was estimated by the food weight difference divided by number of mice in the cage. Nonfasted blood glucose levels were measured monthly consistently at 2PM using a drop of tail blood with a glucometer (CONTOUR®NEXT EZ meter).

### Performance tests

Cognitive function was compared between mouse cohorts using a spatial navigation task (Box maze), a behavioral paradigm which reflects learning and cognitive flexibility^22^. In this task, mice were introduced into a large clear plastic square box with an overhead bright light. The sides of the box were covered with aluminum foil and had floor-level escape holes, seven which were blocked and one which was open leading to a darkened standard mouse cage. Four consecutive trials were given to each mouse with a maximum limit of 120 s and a 30-s resting period in between each trial. Data were recorded as time in seconds to find and enter the escape hole.

Rotarod is a test for coordinated walking ability and was performed as described^23^ using a rotarod apparatus (Rotamax 4/8, Columbus Instruments, Inc.) that allowed mice to walk on a rotating rod with constantly increasing rotation speed. Mice were placed in the lanes of the rotarod with initial rod speed at 0 RPM. The speed was progressively increased by 0.1 RPM/s (0 to 40 RPM over 5 min) or until all mice were dislodged as determined by an infrared sensor. The time in seconds was recorded for three trails with half-hour resting time in between each trial.

Hand grip is a universal measurement used to assess physical competency in older adults as an indicator of frailty. The grip strength test in mice is similar to the hand grip test for people in that it assesses the ability to grip a device with the front paws^24,25^. Mice were positioned horizontally from a grip bar (Columbus Instruments, Inc.) and pulled back slowly and steadily until they released their grip. The test was repeated five times and peak force for the forelimb paws was recorded. Grip strength force was normalized by body weight measured on the testing date, so that the peak force was expressed relative to body weight.

### Geropathology

Mice in all groups were decapitated after euthanasia via 5 min carbon dioxide inhalation approved by Institute/Center (IC) Animal Care and Use Committee (ACUC). Necropsy was performed after euthanasia with a postmortem time of 5 min. Sections of heart, lung, liver and kidney were quick frozen and stored at −80. Sections of the same organs were also fixed in 10% buffered formalin for 48 h then blocked in paraffin wax through University of Washington Department of Comparative Medicine’s Histology Lab. Sections of 4 µm fixed tissues were stained with hematoxylin and eosin for geropathology grading by two board certified veterinary pathologists (D Imai and T Snider) in a blinded manner according to published guidelines^16^. The geropathology scores were tabulated into a composite lesion score (CLS) for each of four major organs—heart, lungs, liver and kidney, and recorded for each animal. The CLS was calculated by adding the severity score (from 1 to 4) of each lesion in an organ for each mouse in a cohort, and then adding the total lesion score of each organ for all mice in a specific cohort and dividing by the number of mice in that cohort. Therefore, the CLS is a standardized score that can be used statistically to compare lesion severity among different cohorts.

### Quantitative real-time PCR

RNA expression analysis was performed as previously described^26^. Briefly, frozen tissues collected at necropsy were homogenized using FastPrep-24 homogenizer (MP Biomedicals) and total RNA was isolated by Trizol extraction according to manufacturer’s specifications (Thermo Fisher). Total RNA was quantified using a Nanodrop spectrophotometer (Thermo Fisher) and 1 μg of total RNA used to generate cDNA via the Transcriptor First Strand cDNA synthesis kit (Roche) according to the manufacturer’s specifications. Gene expression changes were quantified by qRT–PCR reactions using 20 μl reaction volumes and a StepOne thermocycler (Thermo Fisher) with input of 50 ng total RNA per reaction. For each sample, reactions were performed in duplicate. Data were analysed by the ΔΔCt method and expression was normalized to Gapdh.

### Data analysis

Two-tailed student’s t-test was used to compare between results from each treatment cohort. Chi-square tests were used to determine the association between categorical outcomes and treatment cohorts. For geropathology, scoring data was the average lesion score from two pathologists analyzed by cohort using the two-tailed student’s t-test. Mean values with standard error bars (SEM) are presented by group in the figures. Quantitative interval outcomes collected from both pathologists were normalized for central tendency analysis.

A mixed-effects model with random intercept was applied to investigate the qPCR results of senescence associated factor expression^26^. The marginal composite scores were tested through log-transformed relative expression levels. Correlation analysis between treatment cohorts, strains and lesion scores was done by one- and two-way ANOVA. All statistical tests were conducted at 0.05 significance level.

## Results

### Health enhancing effects of the drug cocktail were dose dependent

The main objective of the study was to show that a cocktail of drugs each shown to have anti-aging effects in mice would be more effective in delaying aging phenotypes than any individual drug within the combination. The dose of each drug used in the cocktail was based on published studies^7,14,15^, but since each drug had not been used in this type of combination, there was a need to compare a different dose. Therefore, we tested a cocktail with a half dose of each drug compared to the cocktail with a full dose and control in C57BL/6 mice for three months starting at 20 months of age to 23 months of age. Mice fed the full dose cocktail or half dose cocktail through the 3-month treatment period showed generally similar biological changes (Fig. S1) suggesting the lower dose had a biological effect. Both males and females performed better in the spatial learning task when fed the full dose cocktail or the half dose cocktail chow compared to mice fed the control chow (Fig. S2A). The half dose cocktail was less effective than the full dose cocktail in both sexes performing rotarod grip strength tasks (Fig. S2B, C). The half dose cocktail was not nearly as effective in decreasing age-related lesions as the full dose cocktail, but there were some sex dependent exceptions (Fig. S3). The data showed a pattern of the half dose cocktail that was similar to the full dose cocktail, but less effective, helping validate an effective dose for this study. All subsequent data in this report are from mice fed full dose cocktail, or full dose for each individual drug, added to the chow.

### C57BL/6 mice receiving the drug cocktail showed decreased body weight and improved performance

**Figure 1:**
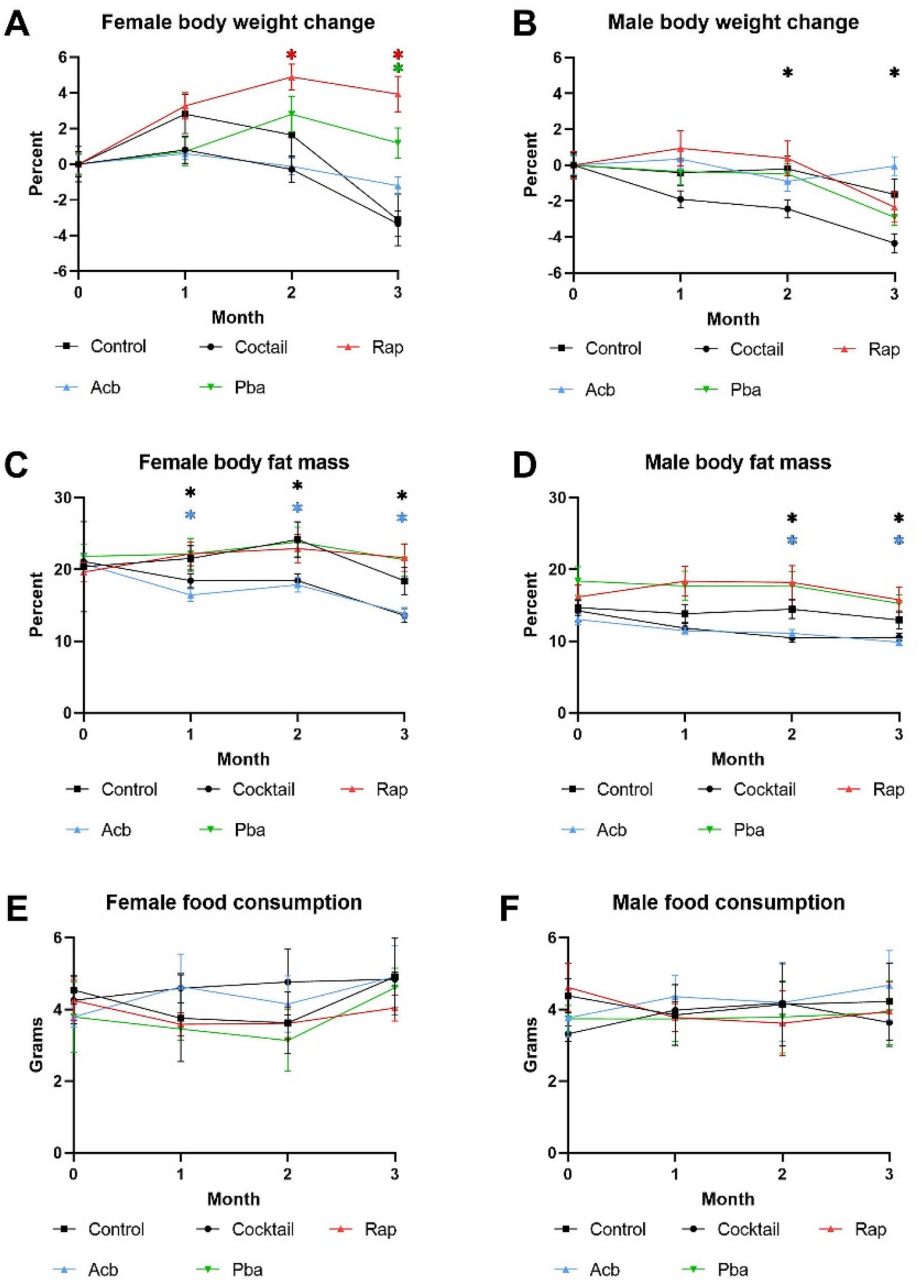
Biological parameters were assessed monthly in C57BL/6 mice fed drug cocktail chow or chow with each individual drug or control for three months starting at 20 months of age. **(A)** Body weight in females, **(B)** body weight in males, **(C)** body fat mass measured by magnetic resonance imaging in females, **(D)** body fat mass measured by magnetic resonance imaging in males, **(E)** food intake measured over 3 days each month for females, **(F)** food intake measured over 3 days each month for males. N = 12–14/cohort. *p ≤ 0.05, ANOVA with Tukey’s post-hoc comparison and two-tailed, unpaired Student’s t-test.

Over the 3-month treatment period, C57BL/6 mice fed the drug cocktail chow showed a decrease in body weight as early as the second month compared to mice fed the control chow, which continued through the third month for males but not females (Fig. 1A,B). For mice receiving individual drug chow, there were sex differences in the Rap and Pba cohorts, with no weight loss in females. The decrease in body fat mass paralleled the decrease in body weight (Fig. 1C,D). We measured 3-day food intake once a month and there was no significant difference in amount of food ingested among the cohorts in either males or females (Fig. 1E,F) indicating caloric intake was not a likely factor in compromising phenotypes effected by the medications in the chow. There were no significant differences in changes in lean muscle mass or blood glucose levels in any of the cohorts over the three-month treatment period (data not shown).

**Figure 2:**
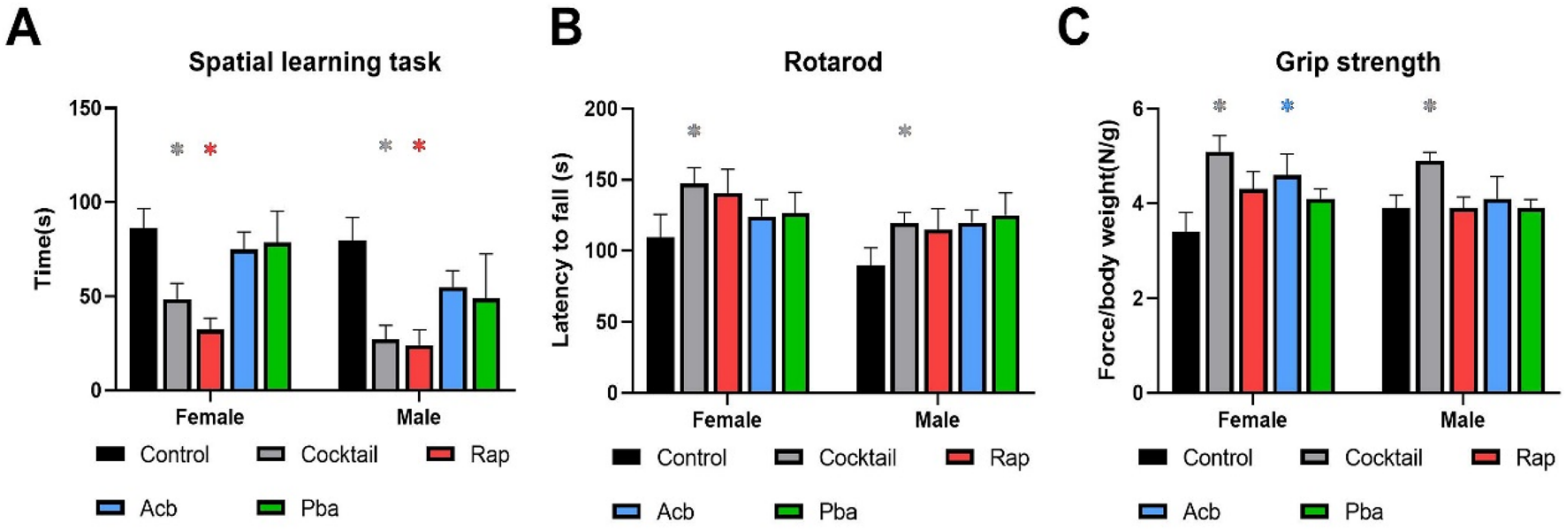
Performance tests were conducted in C57BL/6 mice at 23 months of age, three months after being started on chow containing the drug cocktail or placebo. **(A)** Values for the box maze were standardized times in seconds to find the escape hole in trial 3. **(B)** Values for the rotarod were standardized times in seconds staying on the rotating rod. **(C)** Values for grip strength were standardized force in newtons per body weight measuring the ability to maintain forepaw grip from a parallel meter bar. N = 12–14. *P < 0.05; 2-way ANOVA with Tukey’s post-hoc comparison and two-tailed, unpaired Student’s t-test.

Mice of both sexes fed the cocktail chow were better in finding the escape hole in the spatial learning task compared to mice fed control chow or chow with Acb or Pba (Fig. 2A). However, both male and female mice fed the Rap chow performed just as well as mice fed the cocktail chow. In general, both male and female mice fed the cocktail chow outperformed mice fed individual drug chow in rotarod and grip strength tasks (Fig. 2B,C).

### Decreased severity of age-related lesions occurred in C57BL/6 mice fed drug cocktail chow but not consistently in mice fed chow with each individual drug

**Figure 3:**
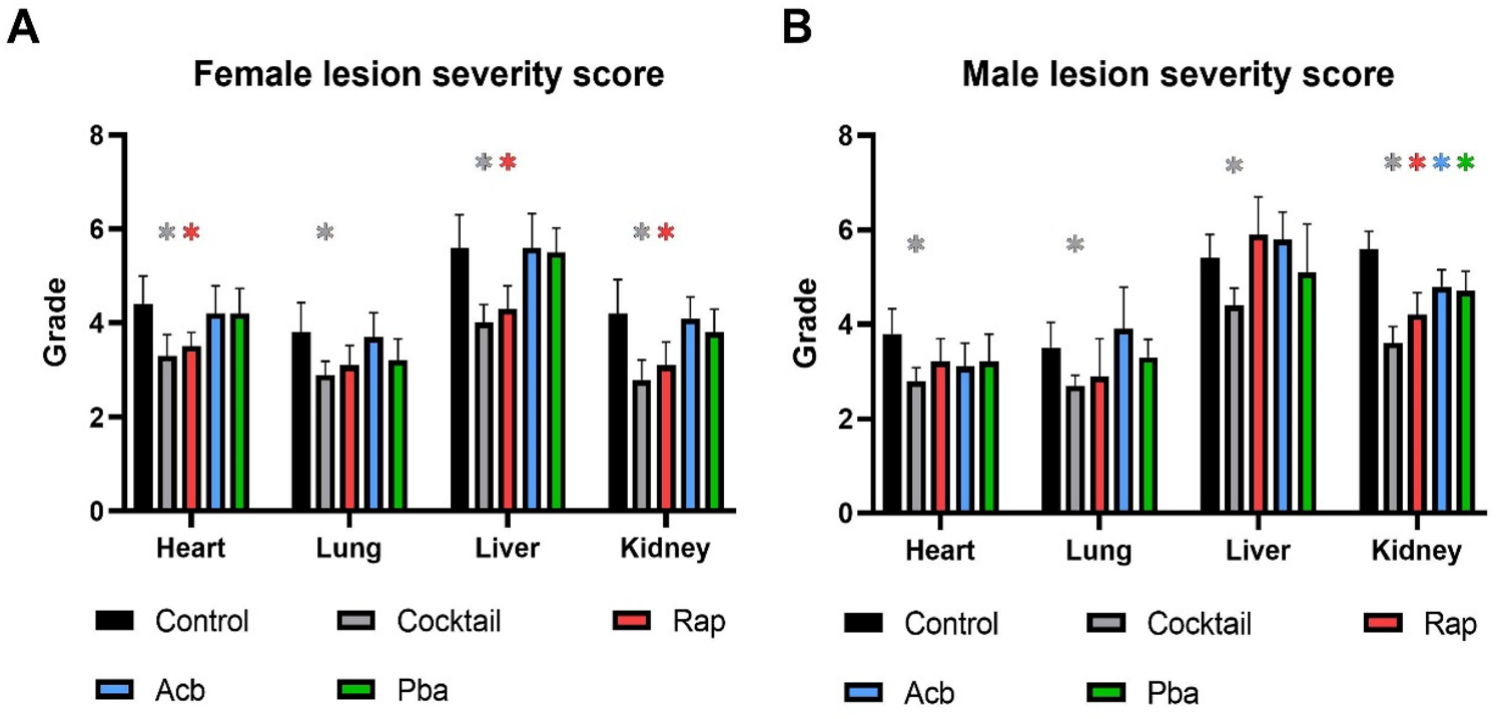
Lesion severity scores were calculated in four major organs from C57BL/6 mice fed chow with a three-drug cocktail or each individual drug for 3 months starting at 20 months of age. **(A)** Female lesion severity scores. **(B)** Male lesion severity scores. N = 12–14/cohort. *P < 0.05; 2-way ANOVA with Pearson chi-square test.

There was a consistent decrease in lesion severity in both males and females fed chow with the drug cocktail (Fig. 3A,B). Chow with individual Rap, Acb or Pba was in general less effective than cocktail chow in decreasing lesion severity, with some exceptions. Rap treatment was effective in decreasing lesion severity in heart, liver, and kidney in females and kidney in males. All three drugs individually were effective in decreasing lesion severity in the kidneys.

**Figure 4:**
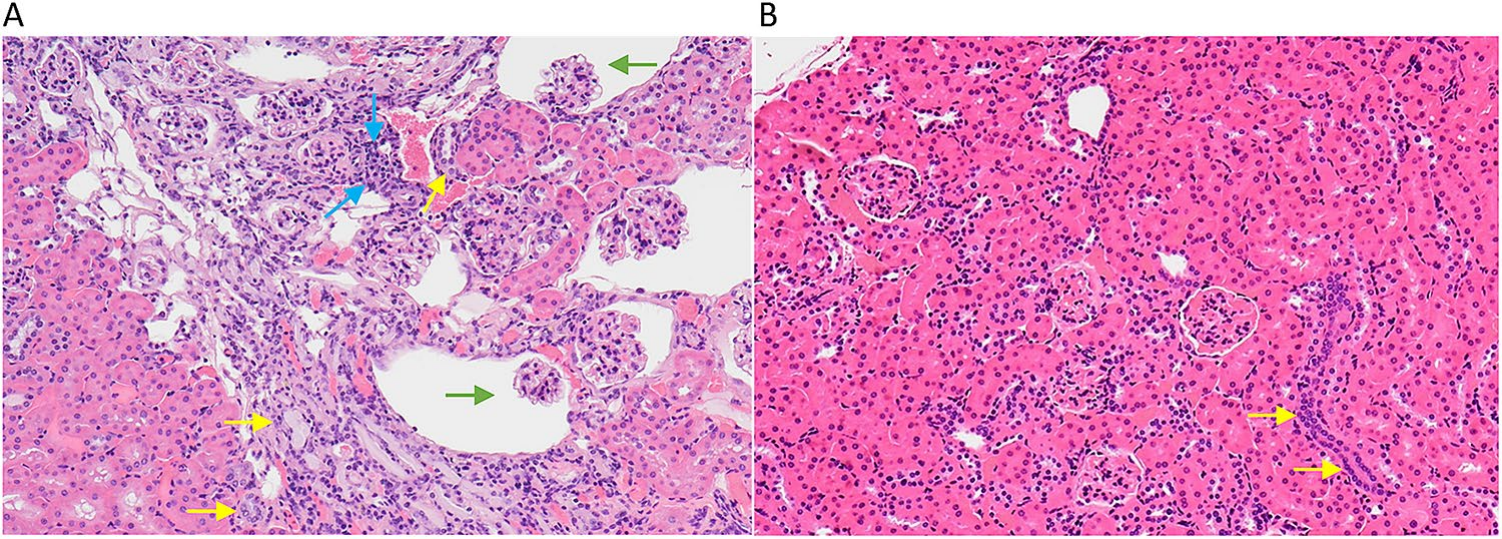
Representative images of H and E-stained kidney sections from mice treated with a drug cocktail or control. **(A)** The control mouse kidney shows moderate to severe aging lesions (scored as a grade 3). Specific lesions include moderate nephropathy characterized by multifocal tubular basophilia (yellow arrows), glomerular atrophy and dilated Bowman’s space (green arrows) and interstitial inflammatory infiltrates (blue arrows). **(B)** The cocktail treated mouse kidney shows fewer and less severe aging changes (scored as a grade 1), characterized by focal tubular basophilia (yellow arrows). Magnification ×200.

The significant decrease in the severity of age-related kidney lesions in mice fed the cocktail chow compared to kidneys from both male and female B6 mice fed the control chow is an example of the impact the drug cocktail had on improving healthy aging in mice. Figure 4A shows a kidney with a moderate to severe geropathology lesion score (grade 3 out of 4) from a B6 female fed the control chow while Fig. 4B shows a kidney with a mild geropathology lesion score (grade 1 out of 4) from a mouse fed the drug cocktail chow.

### The drug cocktail lowered transcriptional levels of inflammatory cytokines in the kidney

**Figure 5:**
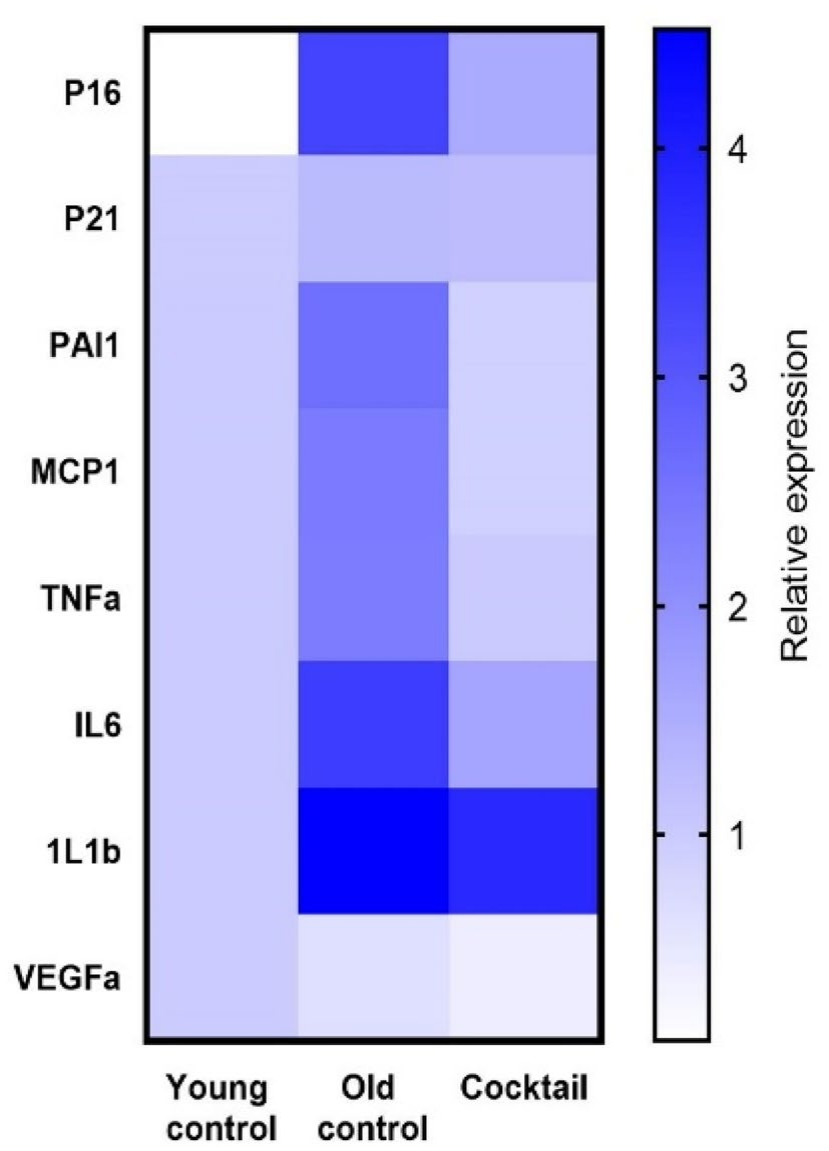
Kidneys from 23-month-old C57BL/6 mice fed the cocktail chow for 3 months showed a general decrease in inflammatory cytokine RNA message by qPCR. Based on the mixed-effect model, which accounts for individual and composite relative expression values, the cocktail diet group showed significantly more anti-inflammatory effect than the control diet group (p < 0.001) and more in line with expression in young mice.

RNA message levels for TNFa, IL6, and MCP-1 in the kidneys from mice fed the cocktail chow were assessed by rtPCR and shown to be significantly lower than in the kidneys from mice fed the control chow (Fig. 5). The lower expression levels were consistent with low expression levels in the kidneys from young untreated mice. Interestingly, IL1b levels were not affected. In addition, p16 but not p21, was moderately decreased suggesting that some aspects of cell cycle regulation may be effected by the cocktail concurrently with downregulation of inflammatory cytokines.

### HET3 mice treated with the drug cocktail generally showed responsive phenotypes

**Figure 6:**
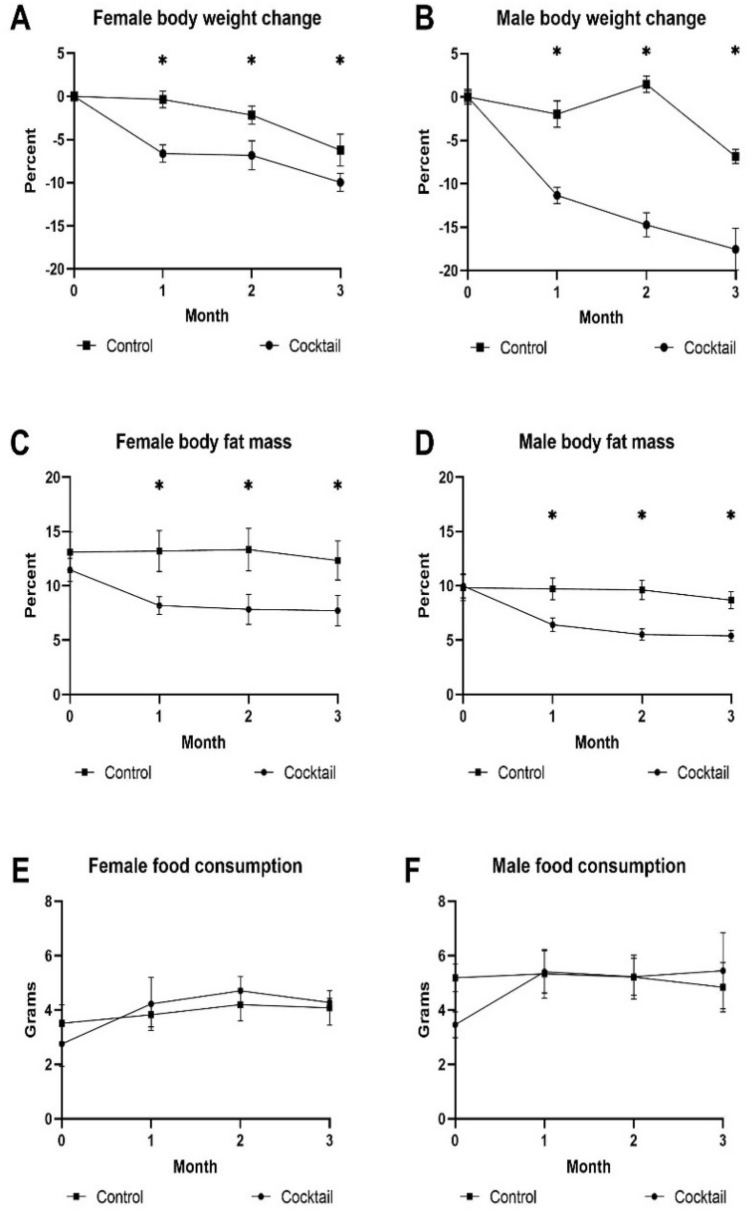
Biological parameters were assessed monthly in HET3 mice fed drug cocktail chow or chow with each individual drug or control for three months starting at 20 months of age. **(A)** Body weight in females, **(B)** body weight in males, **(C)** body fat mass measured by magnetic resonance imaging in females, **(D)** body fat mass measured by magnetic resonance imaging in males, **(E)** food intake measured over 3 days each month for females, **(F)** food intake measured over 3 days each month for males. N = 12–14/cohort. *P < 0.05; two-tailed, unpaired Student’s t-test.

We wanted to see if there were any differences in how the drug cocktail affected age-related phenotypes in HET3 mice, a commonly used strain in aging studies, compared to B6 mice. The change for body weight, body fat mass, and food consumption over three months of treatment is shown in Fig. 6. HET3 males treated with the drug cocktail lost a significant amount of weight over the three-month treatment period in contrast to B6 females, but other biological parameters were similar.

**Figure 7:**
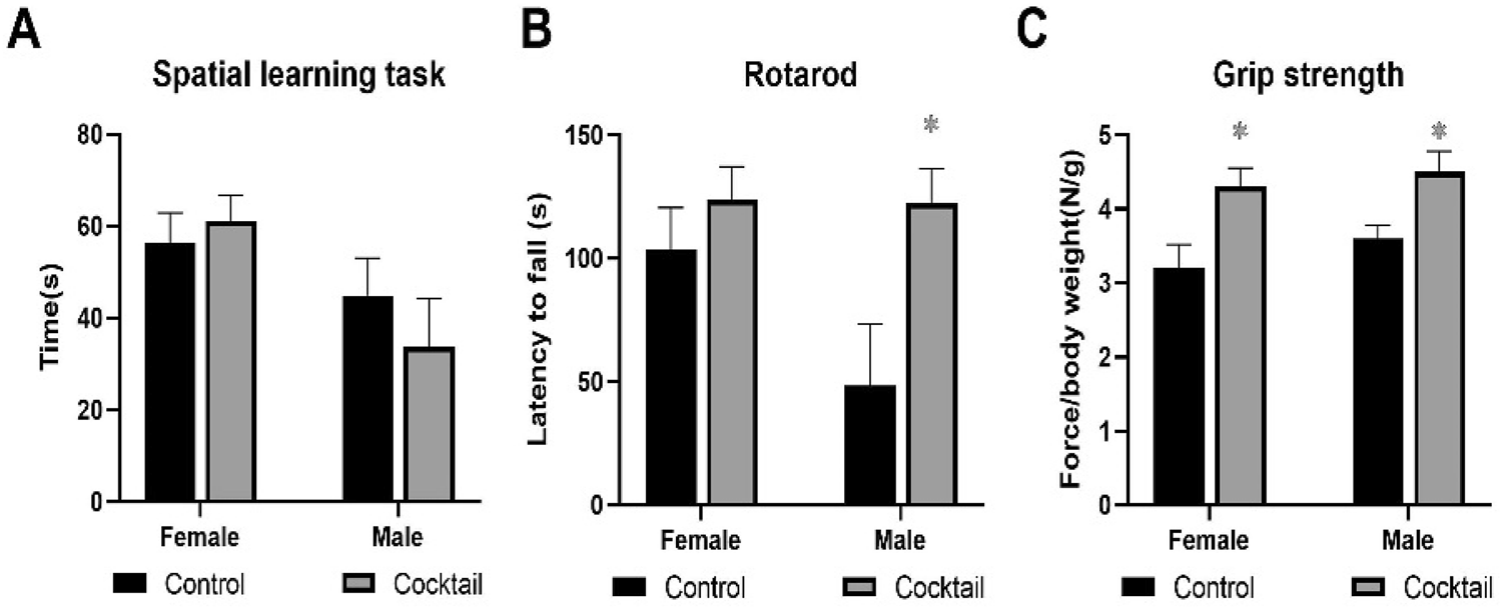
Performance tests were conducted in HET3 mice at 23 months of age, 3 months after being started on chow containing the drug cocktail or placebo. **(A)** Values for the box maze were standardized times in seconds to find the escape hole in trial 3. **(B)** Values for the rotarod were standardized times in seconds staying on the rotating rod. **(C)** Values for grip strength were standardized force in newtons per body weight measuring the ability to maintain forepaw grip from a parallel meter bar. N = 12–14. *P < 0.05; two-tailed, unpaired Student’s t-test.

HET3 males fed the cocktail chow showed a trend for improved cognition, and significantly enhanced performance on the rotarod, while HET3 females fed the cocktail chow showed no improvement in cognition (Fig. 7A) but a trend for improvement in the rotarod task (Fig. 7B). Both sexes fed the cocktail chow showed improved grip strength compared to cohorts fed the control chow (Fig. 7C).

**Figure 8:**
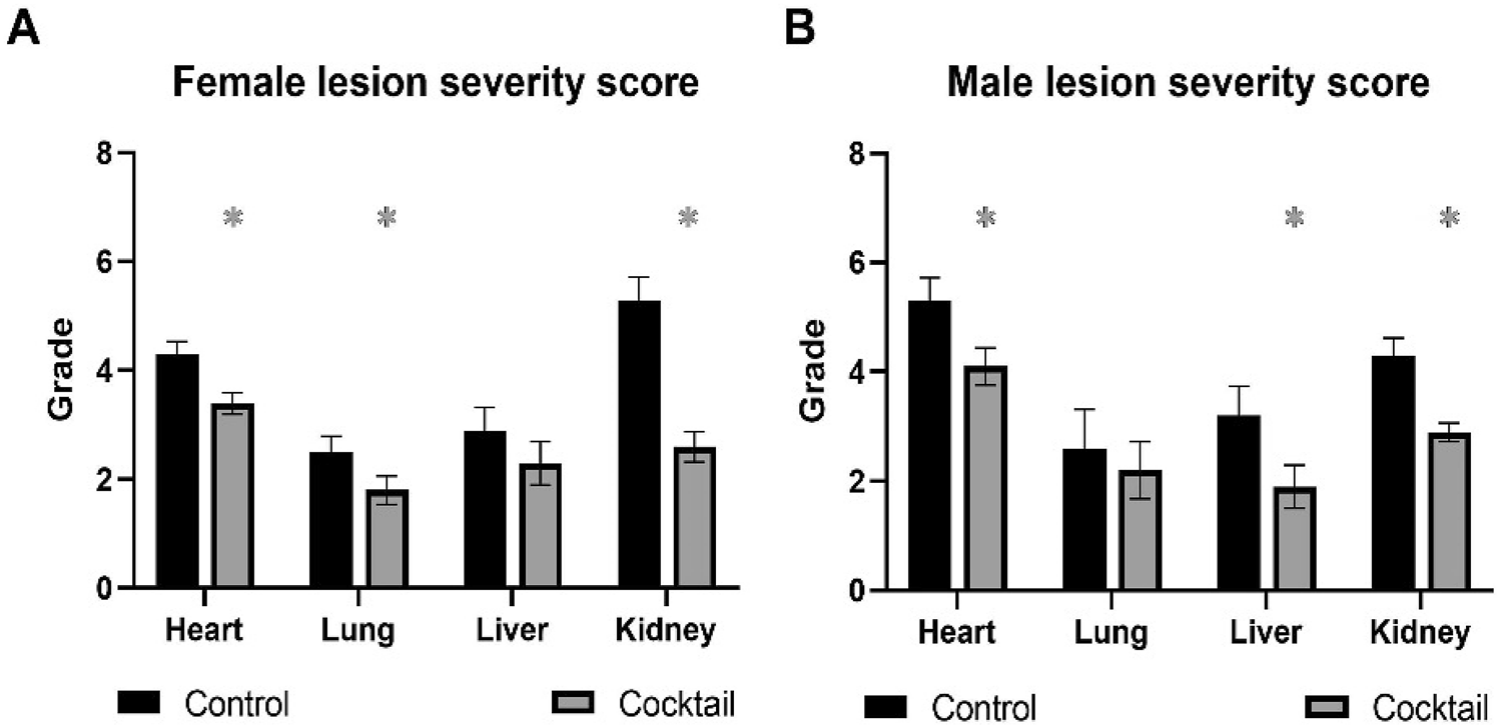
Lesion severity scores were calculated in four major organs from HET3 mice fed chow with a three-drug cocktail or control for 3 months. **(A)** Female lesion severity scores. **(B)** Male lesion severity scores. N = 12–14/cohort. *p ≤ 0.05, Pearson chi-square test.

Age related lesion severity was decreased in organs from HET3 female mice fed the cocktail chow except for liver (Fig. 8A), while HET3 males fed the cocktail chow had decreased lesion severity in organs evaluated except for lungs (Fig. 8B) suggesting there were sex differences in the drug cocktail response to age-related lesions in the HET3 mouse strain.

## Discussion

The drug cocktail of rapamycin (Rap), acarbose (Acb), and phenylbutyrate (Pba) was studied to test the concept that interventions that extend lifespan in mice will result in improvements in multiple aspects of health span, resulting in significant delays in the appearance of poor physiological performance and progression of organ-based pathology. The rationale for testing the drugs in combination as a single cocktail was based on the different but overlapping anti-aging targeting effects of each drug on processes of aging. C57BL/6 (B6) and HET3 4-way cross (HET3) mice, 23 months of age, fed chow containing the cocktail for three months, showed significant strain and sex-dependent improvements in biological and physiological assessments and suppression of age-related lesions compared to mice fed individual drug or control chow.

In order to confirm that the dose of the drugs in the cocktail would be biologically effective over the three-month treatment period in B6 mice, we tested a different dose. B6 mice were fed a cocktail diet containing one-half the dose of each drug compared to the full dose cocktail diet and the control diet. Interestingly, the half-dose drug cocktail was just as effective as the full dose drug cocktail in preventing age-related cognitive impairment, but was less effective in the other physical performance tests. The half-dose cocktail also had no effect on reducing pathological lesions, suggesting again that there is some specificity in the outcomes based on the dose of drugs used. Higher concentrations of the drugs were not tested, so it is not known whether a higher drug dose cocktail would have had a more comprehensive effect. We concluded that the cocktail drug dose (full dose) used in our study was adequate to show significant age-related phenotypic differences with mice treated with the individual drugs or control (no drugs).

The major biological changes that we measured in B6 mice treated with the drug cocktail for 3 months were in body weight and body fat mass. Body weight was decreased by the cocktail in females after one month of treatment and after 2 months of treatment in males. The trend in females was not seen after three months of treatment but was seen as a significant decrease in males suggesting sex differences in metabolic response to the drug cocktail. This observation was further validated by the concurrent decrease in body fat mass in both sexes. Interestingly, B6 female mice treated with Rap or Acb actually gained weight, but males did not adding evidence of the sex dependent differences in metabolic response to the individual drugs. Acb treatment consistently decreased body fat mass in both females and males similar to the drug cocktail. Since there were no significant changes in lean muscle mass in any of the cohorts, changes in body weight could be explained by changes in body fat mass in most instances. Effect of the cocktail diet on fat mass was striking in both strains and appeared to be due to Acb. One of the explanations for these phenotypic responses might be due to the glucoregulatory control effect by Acb, which selectively inhibits carbohydrate metabolism by allowing caloric compensation in food intake^27^. This selective inhibition promotes a similar effect as caloric restriction, which reduces calorie consumption provided by all macronutrients. Previous studies showed that Acb treatment led to lifespan extension principally in male mice^28^. Acb related metabolic pathways were similar in female and castrated males suggesting the sexual dimorphism effect was related to gonadal hormone differences. The sex bias for changes in fat mass were not as apparent in this study where decreases in fat mass occurred in females after one month of treatment with cocktail or Acb and after two months of treatment in males. These decreases continued in both sexes through three months of treatment.

Several tests were conducted to measure physiological performance in B6 mice treated with the drug cocktail or each individual drug. Both males and females fed the cocktail chow showed improved cognitive performance in the spatial learning task. When fed chow with each individual drug, Rap but not Acb or Pba, showed an effect similar to the drug cocktail in both sexes. Rap thus appeared to be the major contributor for the cocktail’s effect on suppressing cognitive impairment. Decreased neuronal activation and imparied cognitive peformance during aging occurs in both humans and rodents. Chronic mTOR attenuation by rapamycin has shown the benefits of restoring deficits in neurovascular coupling response and cerebovascular dysfunction in aging rodent models^29^. Similar effects may be occurring in the current study.

Additional performance tests in B6 mice included rotating rod and grip strength tasks. Both females and males fed chow with drug cocktail performed better in both tasks compared to females and males fed the control chow. In order to see if a partciular drug in the cocktail was a major contributor to effects seen in these two performance tests, mice were fed chow containing each drug, or the cocktail or control. No single drug consistently enhanced performance in both sexes compared to the cocktail. However, female mice fed chow with Acb performed equally well in the grip strength task as females fed chow with the drug cocktail. The fact that this sex-dependent result in strength performance was not seen in the drug cocktail treated mice suggests that the other drugs in the cocktail contributed in some way.

Geropathology assessment of age-related lesions can provide useful information as to how drug treatment can effectively target organs and specific lesions within organs^30^. Data from this study showed a consistently significant decrease in lesion scores of heart, lungs, liver and kidney from both female and male B6 mice fed chow with drug cocktail compared to lesion scores in these organs from mice fed control chow (no drugs). This type of assessment provides an indepth view of how individual organs responded to drug treatment in B6 mice, and based on the data, we can say the drug cocktail was very effective in delaying the progression of age-related pathology in all organs examined. We view this as a vital component of the study since mice were treated for only three months, which suggests that short term cross-sectional studies designed to test drugs for anti-aging effects in mice would benefit by including geropatholgy assessment. When treatment with individual drugs was compared to treatment with the drug cocktail in B6 mice, there were some unexpected sex dependent results. Rap was almost as effective as the drug cocktail in suppressing age-related lesions in females in heart, liver, and kidney, but much less effective in males (kidney only). The other two drugs, Acb and Pba, were not effective in females at all, and were effective in males only in the kidney. These observations suggest that the adminsitration of the three drugs in combination as a cocktail has a major advantage over any individual drug tested in the study. In addition, the fact that age-related lesions in the kidney seemed to be very responsive to drug treatment suggests that geropathology assessment of the kidney should be included in any short term drug study in aging B6 mice.

Based on geropathology observations in the kidney showing extensive inflammatory activity in mice fed control chow (Fig. 4), it was of interest to explore possible transcriptomic changes associated with suppression of inflammation in mice fed the drug cocktail chow. Using qPCR, RNA message levels were detemined for TNFa, IL6, MCP-1, and IL1b in the kidneys from cocktail treated mice and control mice. The lower expression levels of TNFa, IL6, and MCP-1 in cocktail treated mice were consistent with low expression levels in the kidneys from young untreated mice suggesting the drug cocktail delays aging in the kidney partly by anti-inflammatory effects. The fact that RNA message for p16, a gene involved in cell cycle regulation, was also decreased further strengthens this observation. In addition, RNA message for PAI-1 and VEGFa, genes involved in vascular integrity, was decreased in mice fed the drug cocktail chow and is of interest because vascular thrombosis with infarcts (areas of ischemic necrosis) is not uncommon in the kidneys of old B6 mice. These lesions were rarely seen (data not shown) in kidneys from the cocktail treated mice suggesting these genes may be possible anti-aging therapeutic targets in mice.

Observations from this study seen in C57BL/6 (B6) mice are of interest but need to be substantiated in a second mouse strain because of strain variability in drug responsiveness. HET3 4-way cross (HET3) mice are used exclusively by the NIA Intervention Testing Program in lifespan studies to test a large number of drugs^7^, including the three drugs in our cocktail. Therefore, it was considered a useful strain to help validate the effects of the cocktail on aging parameters in B6 mice. The HET3 mice were tested in the same manner, age, and timing as the B6 mice, but only with the drug cocktail compared to control chow. Overall, HET3 mice were responsive to the drug cocktail. However, there were several differences in the manner of the response, especially depending on sex. First, there was a robust decrease in body weight over the three-month treatment period, especially in males. As in B6 mice, this weight loss was reflected in decreases in body fat mass but not lean muscle mass. There were no differences in amount of food consumed so the metabolic changes were instigated by the drug cocktail. Secondly, both female and male HET3 mice fed drug cocktail chow outperformed mice fed control chow in the grip strength task. However, only male mice on the drug cocktail chow showed improved performance in the rotarod task, while neither female nor male HET3 mice fed cocktail chow showed improved learning ability as assessed by the spatial learning task. Third, severity of heart and kidney lesions were consistently decreased in both females and males fed cocktail chow, while severity of liver lesions was not affected by the cocktail in females and severity of lung lesions was not affected by the cocktail in males. As more therapeutic testing is done in mice, more studies are needed to point out strain differences in drug responsiveness. Because we tested both sexes, HET3 mice were helpful in corroborating the drug cocktail as an effective therapeutic approach to delay aging phenotypes.

In conclusion, the drug cocktail was more effective than each individual drug, and a half dose of the drug cocktail in the diet was overall less effective than the full dose cocktail based on biological, physiological and geropathological endpoints. The sex and strain differences partly agree with previous observations in lifespan studies in mice treated with Rap or Acb^7,14^. Interestingly, previous studies also suggest that combining pro-longevity treatments may have beneficial effects on longevity and health span beyond that of each drug individually. This study shows that a combination of three drugs previously shown to enhance lifespan and health span in mice is able to delay aging phenotypes more effectively and more robustly than any individual drug in the cocktail when started at middle age and given for a short period of time.

## Supplementary Information

Supplementary Information.

## Data Availability

All data analyzed during this study are included in this published article.
