# Supplementary material for: Short term treatment with a cocktail of rapamycin, acarbose and phenylbutyrate delays aging phenotypes in mice
Source: Sci Rep. 2022 May 4;12:7300. doi: 10.1038/s41598-022-11229-1 (PMC9067553; doi:10.1038/s41598-022-11229-1)
Supplement: Supplementary file 1 — Supplementary Information. [file 41598_2022_11229_MOESM1_ESM.docx]

**Short term treatment with a cocktail of rapamycin, acarbose and phenylbutyrate delays aging phenotypes in mice**

Zhou Jiang^1^, Juan Wang^2^, Denise Imai^3^, Tim Snider^4^, Jenna Klug^1^, Ruby Mangalindan^1^, John Morton^1^, Lida Zhu^5^, Adam B. Salmon^6^, Jackson Wezeman^1^, Jiayi Hu^7^, Vinal Menon^7^, Nicholas Marka^8^, Laura Neidernhofer^7^, Warren Ladiges^1*^

^1^Department of Comparative Medicine, School of Medicine, University of Washington, Seattle, WA

^2^Biological Sciences Division, Pacific Northwest National Laboratory, **Richland, WA**

^3^Department of Pathology, Microbiology and Immunology, School of Veterinary Medicine, University of California, Davis, CA

^4^ Department of Veterinary Pathobiology, College of Veterinary Medicine, Oklahoma State University, Stillwater, OK

^5^ HD Bioscience Co., Ltd, In vivo pharmacology, China

^6^ San Antonio Sam and Ann Barshop Institute for Longevity and Aging Studies and Department of Molecular Medicine, The University of Texas Health Science Center at San Antonio, Department of Molecular Medicine, South Texas Veterans Health Care System, Geriatric Research Education and Clinical Center, San Antonio, TX

^7^Department of Biochemistry, Molecular Biology, and Biophysics, and Institute on the Biology of Aging and Metabolism, University of Minnesota, Saint Paul, MN

^8^Clinical and Translational Sciences Institute, Biostatistical Design and Analysis Center, University of Minnesota, Minneapolis, MN

***Correspondence.** Warren Ladiges, wladiges@uw.edu

**Supplement**

**LabDiet 5LG6 ingredients.** Whole Wheat, Ground Corn, Ground Oats, Wheat Middlings, Fish Meal, Dehulled Soybean Meal, Soybean Oil, Dehydrated Alfalfa Meal, Corn Gluten Meal, Dicalcium Phosphate, Brewers Dried Yeast, Calcium Carbonate, Menadione Dimethylpyrimidinol Bisulfite (source of Vitamin K), Salt, DL-Methionine, Choline Chloride, Magnesium Oxide, Pyridoxine Hydrochloride, Cholecalciferol, Thiamine Mononitrate, Ferrous Sulfate, Vitamin A Acetate, Biotin, Calcium Pantothenate, Manganous Oxide, Calcium Iodate, Vitamin B-12 Supplement, DL-Alpha Tocopheryl Acetate, Folic Acid, Riboflavin Supplement, Nicotinic Acid, Cobalt Carbonate, Zinc Oxide, Ferrous Carbonate, Copper Sulfate, Zinc Sulfate.

**NIH31 diet ingredients.** Fish meal, soybean meal, alfalfa meal, corn gluten meal, ground whole hard wheat, ground #2 yellow shelled corn, ground whole oats, wheat middlings, Brewers dried yeast, soy oil, salt, dicalcium phosphate, ground limestone, d-calcium and vitamins A, D3, dl alpha-tocopheryl acetate, choline chloride, folic acid, niacin, pantothenic acid, riboflavin, thiamin mononitrate, B12, pyridoxine hydrochloride, and d-Biotin.

**qPCR primer sequences**

Cdkn2a (p16Ink4a) Fwd 5′-CCCAACGCCCCGAACT-3′, Cdkn2a (p16Ink4a) Rev 5′-GCAGAAGAGCTGCTACGTGAA-3′; Cdkn1a (p21Cip1) Fwd 5′-GTCAGGCTGGTCTGCCTCCG-3′, Cdkn1a(p21Cip1) Rev5′-CGGTCCCGTGGACAGTGAGCAG-3′; Mcp1 Fwd 5′-GCATCCACGTGTTGGCTCA-3′, Mcp1 Rev 5′-CTCCAGCCTACTCATTGGGATCA-3′; Tnf Fwd 5′-ATGAGAAGTTCCCAAATGGC-3′, Tnf Rev 5′-CTCCACTTGGTGGTTTGCTA-3′; Il6 Fwd 5′-CTGGGAAATCGTGGAAT-3′, Il6 Rev 5′-CCAGTTTGGTAGCATCCATC-3′;

**Figures**

***
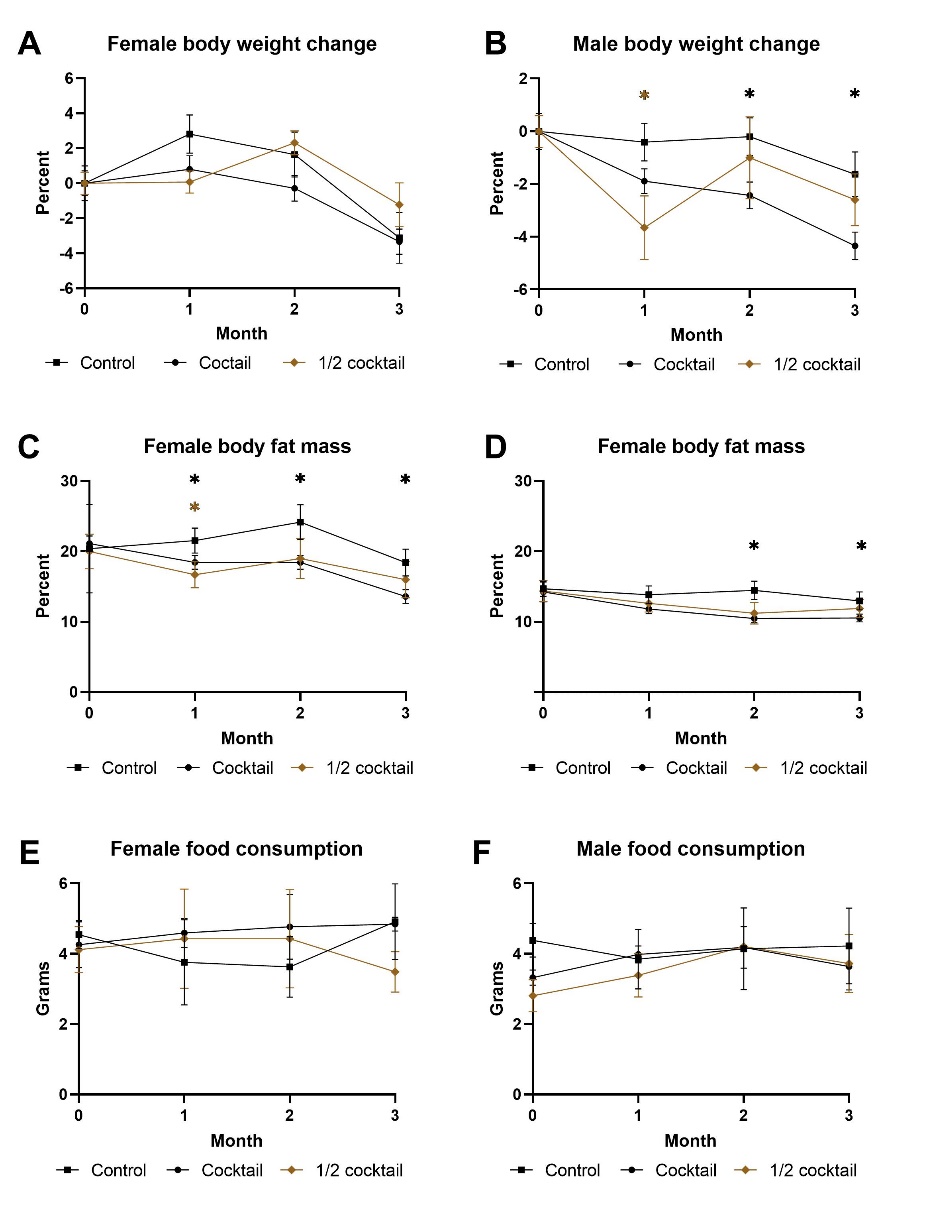
***

**Figure S1.** Biological parameters were assessed monthly in C57BL/6 mice fed drug cocktail chow, a half-dose drug cocktail chow, or control chow for three months starting at 20 months of age. A) Body weight in females, B) Body weight in males, C) Body fat mass measured by magnetic resonance imaging in females, D) Body fat mass measured by magnetic resonance imaging in males, E) Food intake measured over three days each month for females, F) Food intake measured over three days each month for males. N=12-14/cohort. *P < 0.05; two-tailed, unpaired Student’s t-test.


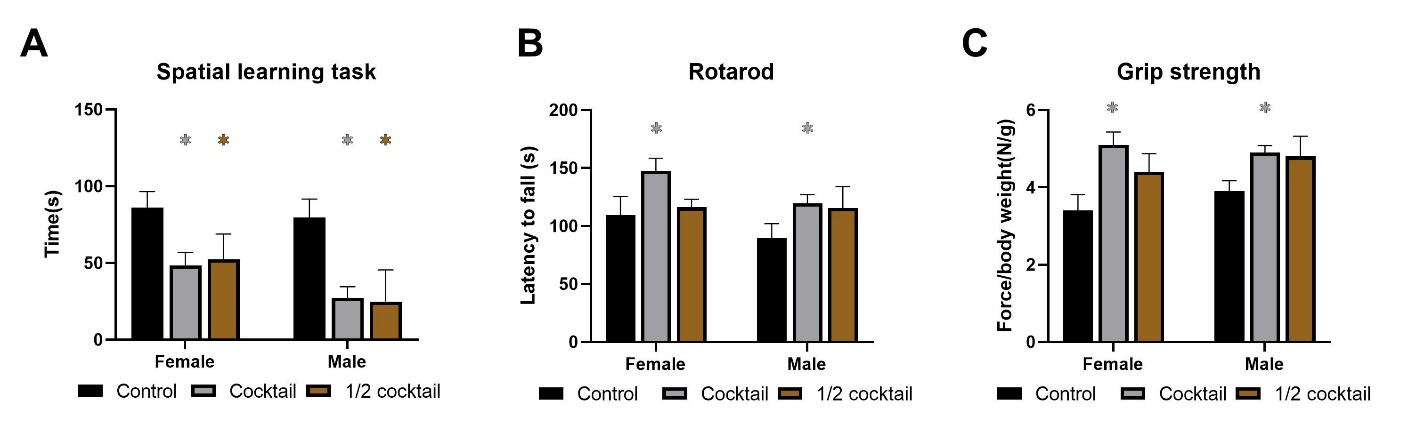


**Figure S2.** Performance tests were conducted in C57BL/6 mice at 23 months of age, three months after being started on chow containing the drug cocktail, a half-dose drug cocktail, or placebo. A) Values for the box maze were standardized times in seconds to find the escape hole in trial 3. C) Values for the rotarod were standardized times in seconds staying on the rotating rod. D) Values for grip strength were standardized force in newtons per body weight measuring the ability to maintain forepaw grip from a parallel meter bar. N = 12-14, *P < 0.05; two-tailed, unpaired Student’s t-test.

**
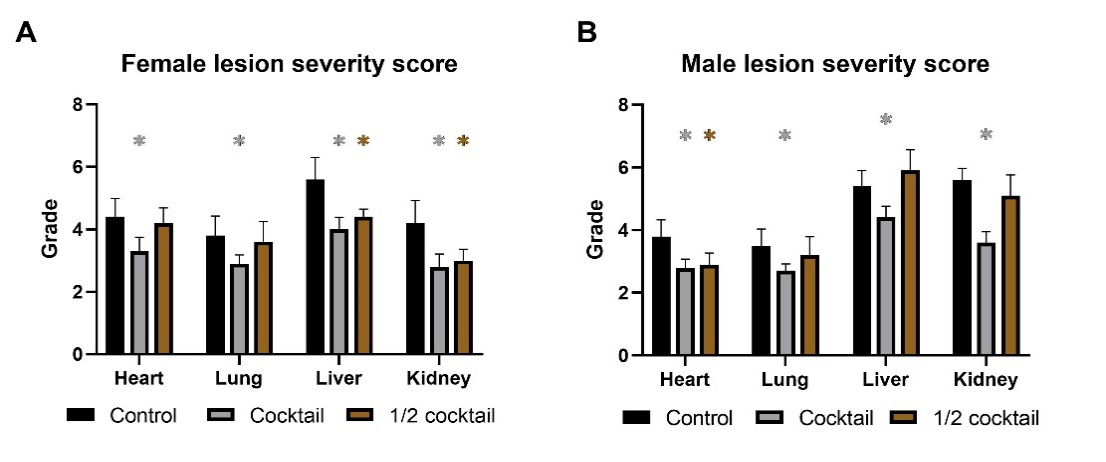
**

**Figure S3.** Lesion severity scores were calculated in four major organs from C57BL/6 mice fed chow with a drug cocktail, a half-dose drug cocktail, or placebo for 3 months starting at 20 months of age. A) Female lesion severity scores. B) Male lesion severity scores. N = 12-14/cohort. *p≤0.05, Pearson chi-square test.
